# Locally advanced leiomyosarcoma of the spleen. A case report and review of the literature

**DOI:** 10.1186/1477-7819-5-135

**Published:** 2007-11-28

**Authors:** Paolo Piovanello, Vincenzo Viola, Giampaolo Costa, Massimiliano Carletti, Andrea Cecera, Fabrizio Turchetta, Roberto Iudicone, Giuseppe Catalano, Antonello Santucci, Franco Recchia, Loredana Fiorillo, Maria Antonella Menichella, Giovanni Baiano

**Affiliations:** 1 Department of General and Thoracic Surgery, "San Giovanni di Dio" Hospital – Fondi (Latina), Italy; 2 Department of Anesthesiology – "San Giovanni di Dio" Hospital – Fondi (Latina), Italy; 3 Operative Room Nursery, "San Giovanni di Dio" Hospital – Fondi (Latina), Italy

## Abstract

**Background:**

Leiomyosarcomas are rare tumours, predominantly localized in the stomach, small intestine and retroperitoneum. Only one case of primary leiomyosarcoma of the spleen is described in human beings in literature.

**Case presentation:**

We report a case of locally advanced primary leiomyosarcoma of the spleen in a 54 year-old woman, diagnosed only after splenectomy, performed with the suspicion of splenic haematoma.

**Conclusion:**

Due to the lack of cases, no specific chemotherapy regimen has been tested to provide a longer survival.

## Background

Leiomyosarcomas are rare tumours, predominantly localized in the stomach, small intestine and retroperitoneum. Only one case of primary leiomyosarcoma of the spleen is described in human beings in literature.

## Case presentation

**Figure 1:**
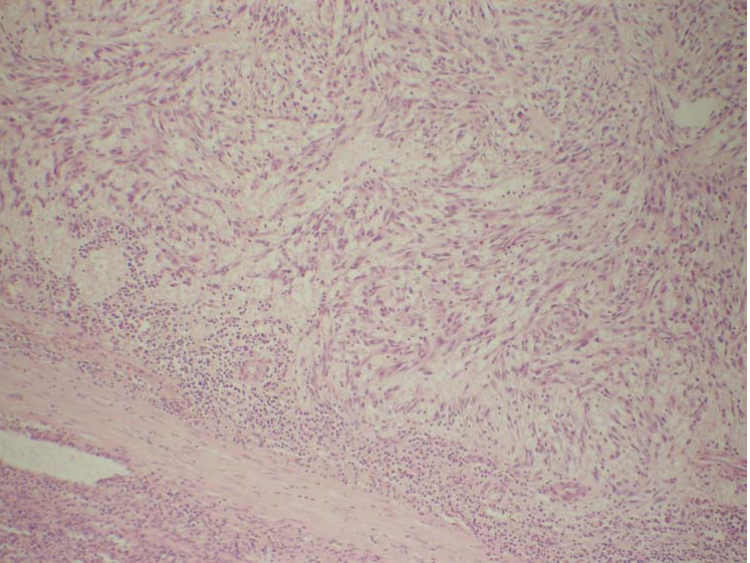
histological finding of the removed spleen (hematoxylin eosin). Non capsulated spindle cells, with oedema and lymphocytic infiltration. No necrosis is present in this field.

**Figure 2:**
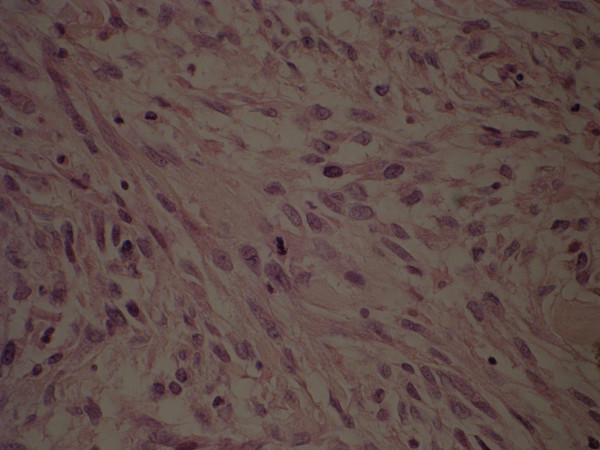
Photomicrograph showing moderate pleomorphism and rare mitosis are present (× 40 hematoxylin eosin).

A 54-year-old Caucasian woman was referred to our department with a history of left-sided abdominal pain, lasting about 8 months. No other signs or symptoms were present at the moment of observation. Past medical history revealed no significant medical problems. Hysterectomy for endometriosis had been performed ten years before admission. No history of carcinogenic exposure was reported. At admission physical examination revealed a palpable spleen 4 cm below the left costal margin. No hepatomegaly was noted. Laboratory exams showed no alterations, except for mildly elevated lactic acide dehydrogenase (LDH). Chest X-ray showed a normal mediastinum and lung parenchyma. Abdominal US revealed, in the superior portion of the spleen, multiple target-shaped focal lesions, one of these with a large anechoic fluid-filled area. CT scan showed a large, inhomogeneous mass of the spleen, of 10 × 7 cm in diameter. This mass was isodense, without enhancement after contrast injection, and had low density fluid-filled areas. The liver parenchyma was normal and no evidence of retroperitoneal lymphadenopathy was found. Emergency laparotomy was performed, due to the suspicion of splenic haematoma. Neither obvious metastases nor hepatic lesions or abdominal fluid were found at laparotomy. An enormous mass of superior portion of the spleen was found, with infiltration of left suprarenal gland and of a portion of diaphragm. Due to the local dissemination a splenectomy and left surrenalectomy were performed, along with a partial diaphragm resection, with a free resection margin of about two centimetres. Splenic vessel lymphadenectomy was also performed. There were no postoperative problems and the patient was discharged on 7th postoperative day. Two different Institution was necessary to obtain the definitive diagnosis. Histological examination showed a spleen of 11 × 5 × 5 cm, with a soft dark lesion, of 10 cm diameter, in the upper pole. Gross examination showed no haemorrhage or necrosis. Microscopic examination revealed a non capsulated spindle and polyhedral cell proliferation along with multiple foci of cellular necrosis. Tumor cells, with pleomorphic and atypic vesicular nuclei, showed marked pleomorphism and rare mitotic figures (Figure 1, 2). Immunohistochemistry showed that the neoplastic cells were positive for smooth muscle actin and for h-caldesmon and negative for CD34, calponin, FVIII-associated antigen, S100, EMA and cytokeratin AE1 AE3. There was left suprarenal gland and diaphragm involvement. The examined lymph nodes had no neoplastic infiltration. Diagnosis of locally advanced primary leiomyosarcoma of the spleen was made (Figure 1).

The patient underwent specific chemotherapic regimen (5 cycles) with epirubicin 90 mg (days 1 and 2) and ifosfamide 2700 mg (days 1 – 5) every 21 days, with no significant adverse reactions.

Actually, 21 months after operation, the patient is alive and totally disease-free.

## Discussion

Leiomyosarcoma is a rare tumour, predominantly localized in stomach, small intestine and retroperitoneum [1]. It is also described in genitourinary tract and inferior vena cava [2]. The aetiology is unknown, and the clinical presentation may be variable. The correct diagnosis is usually made at a late stage, with frequent metastatic spread to the liver (the most frequent), skin, bone, lung, brain and soft tissue [1,3]. For this reason the prognosis of this tumour is poor. The most important criterion of malignancy are: number of mitotic figures present [4], high cellularity, atypia, large size and distant diffusion [1]. The most important prognostic factors for primary gastrointestinal leiomyosarcomas have been shown to be histologic grade, local invasiveness, and extent of resection [5]. A suspicious diagnosis can be obtained with US, CT scan and MRI with gadolinium, although diagnosis can be made only at laparotomy, after pathological examination of the removed specimen. The differential diagnosis include haemangioma, littoral cell angioma, lymphangioma, lymphoma, angiosarcoma, other rare sarcomas and metastases [6]. In our patient, diagnosis of spontaneous splenic haematoma or lymphoma was initially considered, due to the lack of other sign or symptoms. Primary human splenic localization of leiomyosarcoma, although described in canine spleens in 16 cases [7,8], is described in only one case in literature [3]. The other case described demonstrated the poor prognosis of these tumours with early metastatic spreading. In our patient neither haematogenous spread nor lymph node involvement was seen, but only local infiltration of suprarenal left gland and diaphragm. The surgery was performed with a curative intent. In the other case splenectomy didn't seem to prevent metastatic diffusion. Due to the lack of cases, no specific chemotherapy regimen has been tested to provide a longer survival. However the use of chemotherapy regimen with activity in treating metastatic soft tissue sarcoma should be offered to these patients.

## Conclusion

Leiomyosarcoma is a rare neoplasm, with a poor prognosis. Along with this case we have described, only one case of splenic localization is reported in literature. The diagnosis can be made only at laparotomy, after pathological examination of the removed specimen; US, CT scan and MRI can be useful in suspecting diagnosis. Splenectomy performed in early stage, without rupture, seems to improve survival. No specific chemotherapy regimen has been tested for this neoplasm, but adjuvant therapy should be offered to these patients, even if only for palliation.

## Competing interests

The author(s) declare that they have no competing interests.

## Authors' contributions

PP, preperation of the draft manuscript, VV, GC, MC, AC, Searching of the literature and helped in preperation of draft FT, RI, GC, AS, FR, LF, MAM, Helped in preperation of manuscript GB final revision of the manuscript.

All authors read and approved the final manuscript.
